# Cancer risk by combined levels of YKL-40 and C-reactive protein in the general population

**DOI:** 10.1038/bjc.2011.501

**Published:** 2011-11-17

**Authors:** K H Allin, S E Bojesen, J S Johansen, B G Nordestgaard

**Affiliations:** 1 Department of Clinical Biochemistry, Herlev Hospital, Copenhagen University Hospital, Herlev Ringvej 75, Herlev 2730, Denmark; 2 Faculty of Health Sciences, University of Copenhagen, Blegdamsvej 3B, København N 2200, Denmark; 3 The Copenhagen City Heart Study, Bispebjerg Hospital, Copenhagen University Hospital, Bispebjerg Bakke 23, København NV 2400, Denmark; 4 Department of Medicine and Oncology, Herlev Hospital, Copenhagen University Hospital, Herlev Ringvej 75, Herlev 2730, Denmark

**Keywords:** biomarker, inflammation, risk prediction

## Abstract

**Background:**

YKL-40 and C-reactive protein (CRP) are biomarkers that may reflect cancer-related subclinical inflammation. We assessed elevated YKL-40 and CRP levels as combined risk predictors for cancer.

**Methods:**

We measured plasma YKL-40 and CRP at baseline in 8706 individuals from the Danish general population.

**Results:**

Hazard ratio (HR) of gastrointestinal cancer for a doubling of YKL-40 levels was 1.37 (95% CI: 1.17–1.61) and indifferent to adjustment for CRP levels. Hazard ratio of lung cancer for a doubling of CRP levels was 1.35 (1.17–1.56) and indifferent to adjustment for YKL-40 levels. Compared to individuals with both low CRP (<1.7 mg l^−1^) and YKL-40 (<154 *μ*g l^−1^), individuals with high YKL-40 but low CRP had an HR of gastrointestinal cancer of 3.36 (1.70–6.64), whereas individuals with high CRP but low YKL-40 had an HR of lung cancer of 2.19 (1.24–3.87). The area under the receiver operating characteristic (ROC) curve was 0.68 for the ability of YKL-40 to predict gastrointestinal cancer and 0.67 for the ability of CRP to predict lung cancer.

**Conclusion:**

Elevated YKL-40 levels are associated with increased risk of gastrointestinal cancer, independently of CRP levels, whereas elevated CRP levels are associated with increased risk of lung cancer, independently of YKL-40 levels.

C-reactive protein (CRP) is a well-known acute-phase reactant that is produced in the liver in response to elevated cytokine levels after an inflammatory stimulus (Pepys and Hirschfield, 2003; Johnson, 2006). Individuals with slightly elevated CRP levels from 2 to 3 mg l^−1^ and above have an increased risk of cardiovascular disease, but also of several other diseases and all-cause mortality (Erlinger *et al*, 2004; Il’yasova *et al*, 2005; Gunter *et al*, 2006; Otani *et al*, 2006; Siemes *et al*, 2006; Trichopoulos *et al*, 2006; Allin *et al*, 2009; Nordestgaard, 2009; Marott *et al*, 2010; Zacho *et al*, 2010a, 2010b). YKL-40 is a newly discovered plasma protein that – like CRP – is elevated during inflammatory conditions and cancer (Cintin *et al*, 1999; Nordenbaek *et al*, 1999; Vos *et al*, 2000; Ostergaard *et al*, 2002; Johansen *et al*, 2004, 2009b). However, whereas CRP is produced in the liver, YKL-40 is produced at the site of pathology by different cells, including cancer cells and cancer-associated macrophages (Junker *et al*, 2005; Johansen *et al*, 2009b).

Chronic inflammation and cancer are associated: many solid cancers arise at sites of chronic inflammation and many solid cancers induce themselves an inflammatory response in the tumour microenvironment (Balkwill and Mantovani, 2001; Coussens and Werb, 2002; Colotta *et al*, 2009). Irrespective of the origin of the cancer-related inflammation, it is likely to result in elevated plasma levels of inflammatory proteins, which may therefore act as biomarkers of cancer. Numerous inflammatory biomarkers exist, but as we aimed to examine both the central and the peripheral response to inflammation, we chose to focus on a combination of an acute-phase protein produced centrally in the liver, that is, CRP, and a protein produced at the site of the inflammation itself, that is, YKL-40. Presently, it is unknown whether CRP and YKL-40 potentially could complement each other in cancer risk prediction.

In this observational cohort study of the general population, we did not seek to evaluate the aetiological aspects of an association between CRP, YKL-40, and cancer risk, but rather considered CRP and YKL-40 as biomarkers reflecting cancer-related subclinical inflammation. Thus, we first examined whether elevated levels of YKL-40 and CRP are mutually independent risk predictors for gastrointestinal, lung, and any cancer. Second, we examined whether YKL-40 and CRP levels combined better predict these risks than either one alone. And finally, we examined the predictive ability of CRP and YKL-40 by using receiver operating characteristics (ROC) curves. For these purposes, we used a prospective study of the Danish general population, the Copenhagen City Heart Study, in which plasma levels of both YKL-40 and CRP were measured at baseline in 8706 individuals who were followed for up to 18 years (Allin *et al*, 2009; Johansen *et al*, 2009a).

## Materials and methods

### Participants

We studied individuals from the Copenhagen City Heart Study, which is a prospective cohort study of the Danish general population (Schnohr *et al*, 2002; Nordestgaard *et al*, 2007). It was initiated in 1976–1978, with follow-up examinations in 1981–1983, 1991–1994, and 2001–2003. Participants in this study are from the 1991–1994 examination, where 16 563 individuals were invited and 10 135 individuals participated. Data collection from the 1991–1994 examination included a self-administered questionnaire, which was reviewed by an investigator at the day of attendance, a physical examination, and blood samples for biochemical analyses. Plasma samples were frozen at −80°C and were used for measurement of CRP and YKL-40 as described below. Height and weight were directly measured at the physical examination, whereas information about covariates such as smoking and alcohol consumption were obtained from the questionnaires. We excluded individuals without a measurement of CRP or YKL-40 due to unavailability of blood (*n*=1259). Furthermore, we excluded individuals with cirrhosis (*n*=105) (cirrhosis distorts levels of both CRP and YKL-40) and with incomplete information about smoking, alcohol consumption, or body mass index (*n*=65). Thus, we included 8706 individuals aged 21–93 years. Follow-up time for incident cancer began at blood sampling and ended at occurrence of cancer, death, emigration, or May 2009, whichever came first. We excluded individuals with a diagnosis of lung cancer (*n*=18), gastrointestinal cancer (*n*=83), or any cancer (*n*=444) before blood sampling from the analysis of the respective cancer types. Mean follow-up period was 13 years (range=0–18 years). Follow-up was 100% complete, that is, we did not loose track of even a single individual.

### Outcome measures

Cancer diagnoses from 1943 through May 2009 were obtained from the National Danish Cancer Registry (Storm, 1988, 1991; Storm *et al*, 1997) and from the National Danish Patient Registry. Diagnoses were classified according to the World Health Organization International Classification of Diseases, Seventh revision (ICD-7) or Tenth revision (ICD-10). The lung cancer diagnosis included cancer of the bronchies and lungs (98% of cancers in the lung cancer category) and cancer of pleura and mediastinum (2% of cancers in the lung cancer category). The gastrointestinal cancer diagnosis included cancer of the oral cavity, pharynx, oesophagus, stomach, small intestine, liver, biliary tract, pancreas, colon, rectum, and anus. The diagnosis of any cancer included any cancer diagnosis, except for non-melanoma skin cancer. Dates of deaths were obtained from the National Danish Civil Registration System.

### Ethics

The ethical committee of Copenhagen and Frederiksberg, Denmark approved the study (KF-100.2039/91). All participants gave written informed consent.

### CRP and YKL-40 analysis

We determined the CRP and YKL-40 levels in the plasma stored at −80°C for 12–15 years. C-reactive protein was measured by a high-sensitivity turbidimetry assay (Dako, Glostrup, Denmark), assessed daily for precision (Allin *et al*, 2009). YKL-40 was measured in duplicates by a commercial two-site sandwich-type enzyme-linked immunosorbent assay (Quidel Corporation, San Diego, CA, USA) (Harvey *et al*, 1998; Johansen *et al*, 2009a), assessed daily for precision.

### Statistical analysis

We analysed data using the STATA statistical software (version 10.1; StataCorp, College Station, TX, USA), and a two-sided *P*-value <0.05 was considered statistically significant. We used logarithmic base 2 transformations of the CRP and YKL-40 levels to estimate risk of cancer for a doubling of CRP and YKL-40 levels; we chose this approach rather than an earlier one with YKL-40 in 10 years age percentile categories (Johansen *et al*, 2009a), because we then used the same statistical approach for CRP and YKL-40 and because our use of age as the time scale in Kaplan–Meier curves and Cox proportional hazards regression models (see below) results in an optimal and precise adjustment for age, which makes the stratification by 10 years age groups redundant. Furthermore, we divided individuals into four categories based on of their CRP and YKL-40 levels. On the basis of previous knowledge, we used the median as cutoff value for CRP levels (1.7 mg l^−1^) and the 90% percentile as cutoff value for YKL-40 levels (154 *μ*g l^−1^) (Allin *et al*, 2009; Johansen *et al*, 2009a). Thus, four categories were generated: CRP <1.7 mg l^−1^ and YKL-40 <154 *μ*g l^−1^, CRP ⩾1.7 mg l^−1^ and YKL-40 <154 *μ*g l^−1^, CRP <1.7 mg l^−1^ and YKL-40 ⩾154 *μ*g l^−1^, and CRP ⩾1.7 mg l^−1^ and YKL-40 ⩾154 *μ*g l^−1^.

We used the Kaplan–Meier method to plot cumulative incidence of cancer against age and used the log-rank test to test for differences between categories. We used Cox proportional hazards regression models to estimate hazard ratios (HRs) of cancer as a function of CRP and YKL-40 levels. To automatically adjust for age, we used left truncation and age as time scale. In addition, HRs were adjusted for sex, smoking status (never, former, or current smoker), smoking dose (cigarettes or equivalent per day), alcohol consumption (women: ⩽168 or >168 g per week; men: ⩽252 or >252 g per week), and body mass index (<18.5, 18.5–24.9, 25–29.9, or ⩾30.0 kg m^−2^). To examine whether elevated CRP and YKL-40 levels are mutually independent risk predictors, we used likelihood ratio tests to compare statistical models including one or both biomarkers. The likelihood ratio test comparing the model including only CRP levels with the model that includes both CRP and YKL-40 levels evaluated whether the model with YKL-40 levels had more ability to predict/explain cancer risk than the model without. Statistical significance of this test means that elevated YKL-40 levels are a risk predictor, independent of CRP levels. Likewise, the likelihood ratio test comparing the model including only YKL-40 levels with the model that includes both YKL-40 and CRP levels evaluated whether the model with CRP levels had more ability to predict/explain cancer risk than the model without. Statistical significance of this test means that elevated CRP levels are a risk predictor, independent of YKL-40 levels.

We tested the Cox proportional hazards assumption graphically. Any indication of non-conformity to proportional hazards assumptions led to further examination with a test based on Schoenfeld residuals. We detected no major violations. For logarithmic base 2 (log 2) transformations of CRP and YKL-40 levels, we assumed linearity between log 2 (CRP levels), respectively, log 2 (YKL-40 levels) and the log-hazards of cancer. We tested this assumption by computing a likelihood ratio test comparing a quadratic model to a linear model and detected no signs of nonlinearity.

The predictive ability of CRP and YKL-40 in the prediction of lung cancer, gastrointestinal cancer, and any cancer was estimated as the area under the curve from ROC curves. Sensitivity was calculated as the number of true positives/(number of true positives+false negatives) and specificity was calculated as the number of true negatives/(number of true negatives+false positives).

## Results

**Figure 1:**
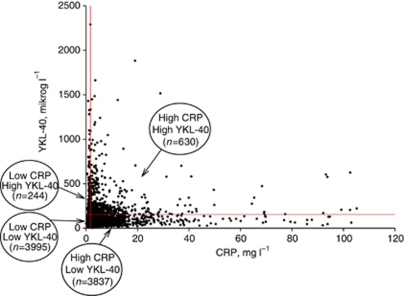
YKL-40 levels by CRP levels in 8706 individuals from the general population. The horizontal red line depicts the median CRP level=1.7 mg l^−1^. The vertical red line depicts the 90% percentile for YKL-40 levels=154 *μ*g l^−1^. From these values, four categories were generated based on combinations of high and low levels of CRP and YKL-40. The colour reproduction of this figure is available at *British Journal of Cancer* online.

Characteristics of participants at blood sampling are listed in Table 1. Figure 1 demonstrates a different distribution of CRP and YKL-40 levels, that is, some individuals have high CRP levels but low YKL-40 levels, and *vice versa*. Hazard ratios of lung cancer, gastrointestinal cancer, and any cancer for a doubling of CRP or YKL-40 levels were attenuated with increasing follow-up time (Supplementary Figure 1). In consequence, we censored individuals at 5 years after blood sampling when calculating HRs of incident cancer. Thus, HRs presented below are HRs of cancer that occurred within the first 5 years after blood sampling. Hazard ratios of cancer that occurred within the full period of follow-up are presented in Supplementary Table 1. Likewise, data on predictive ability of CRP and YKL-40 are based on cancers that occurred within the first 5 years after blood sampling.

### Lung cancer

**Figure 2:**
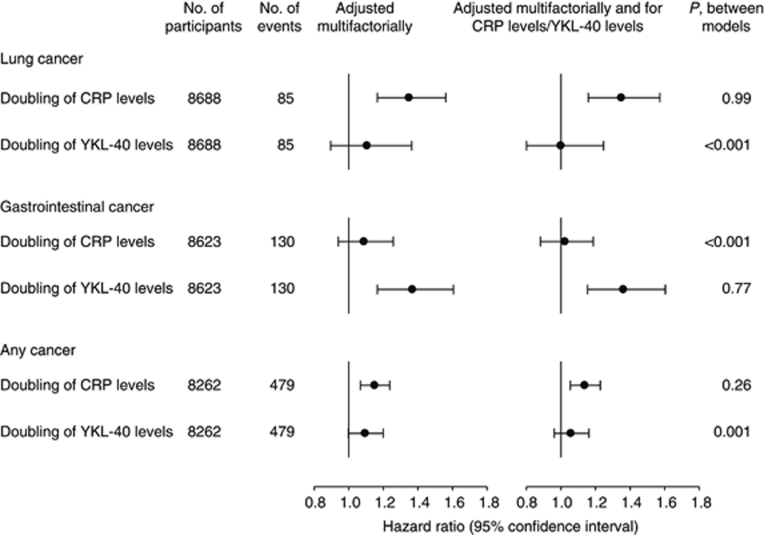
Risk of cancer by CRP or YKL-40 levels in the general population. Multifactorial adjustment included age, sex, smoking, alcohol consumption, and body mass index. *P*-values from likelihood ratio tests examine whether inclusion of YKL-40 levels/CRP levels in the statistical model improves the fit of the statistical model to the data.

The multifactorially adjusted HR of lung cancer for a doubling of CRP levels was 1.35 (95% CI: 1.17–1.56), while further adjustment for YKL-40 levels resulted in a similar HR of 1.35 (1.16–1.57) (Figure 2; likelihood ratio test between models, *P*=0.99). The corresponding HR for a doubling of YKL-40 levels was 1.10 (0.90–1.36), while further adjustment for CRP levels resulted in an HR of 1.00 (0.80–1.25) (Figure 2; *P*<0.001). This means that elevated CRP levels are a risk predictor for lung cancer, independent of YKL-40 levels.

**Figure 3:**
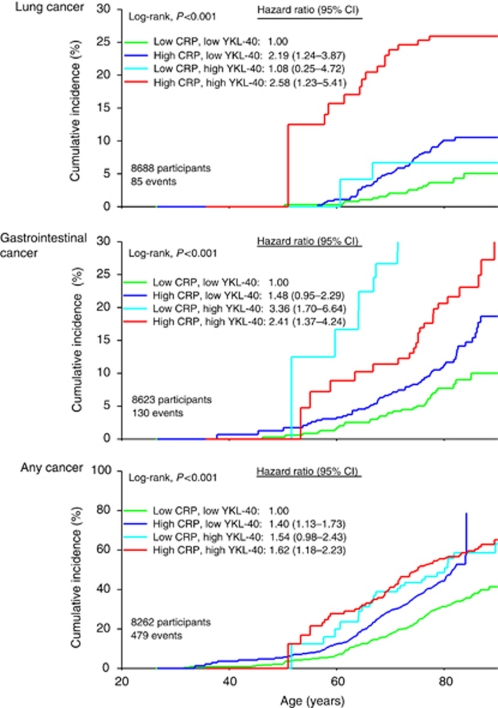
Cumulative incidence of cancer by CRP and YKL-40 levels in the general population. Green line: CRP <1.7 mg l^−1^, YKL-40 <154 *μ*g l^−1^; blue line: CRP ⩾1.7 mg l^−1^, YKL-40 <154 *μ*g l^−1^; cyan line: CRP <1.7 mg l^−1^, YKL-40 ⩾154 *μ*g l^−1^, and red line: CRP ⩾1.7 mg l^−1^, YKL-40 ⩾154 *μ*g l^−1^. Hazard ratios were multifactorially adjusted for age, sex, smoking, alcohol consumption, and body mass index. CI=confidence interval. The colour reproduction of this figure is available at *British Journal of Cancer* online.

The cumulative incidence of lung cancer as a function of age increased with increasing levels of CRP and YKL-40 (Figure 3; log-rank, *P*<0.001). Multifactorially adjusted HRs of lung cancer were 2.19 (1.24–3.87) for individuals with CRP ⩾1.7 mg l^−1^ and YKL-40 <154 *μ*g l^−1^, 1.08 (0.25–4.72) for individuals with CRP <1.7 mg l^−1^ and YKL-40 ⩾154 *μ*g l^−1^, and 2.58 (1.23–5.41) for individuals with CRP ⩾1.7 mg l^−1^ and YKL-40 ⩾154 *μ*g l^−1^ compared to individuals with CRP <1.7 mg l^−1^ and YKL-40 <154 *μ*g l^−1^ (Figure 3).

**Figure 4:**
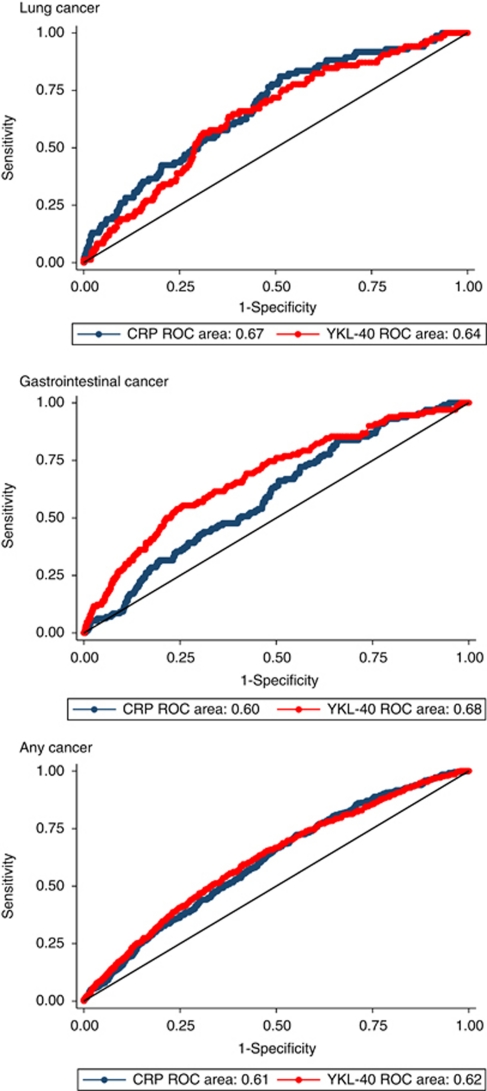
Receiver operating characteristic (ROC) curves illustrating the capacity of CRP and YKL-40 to predict lung cancer, gastrointestinal cancer, and any cancer. ROC area=area under the ROC curve.

The sensitivity and specificity of CRP ⩾1.7 mg l^−1^ was 79 and 49% (Table 2). The corresponding values for YKL-40 ⩾154 *μ*g l^−1^ was 19 and 90% (Table 2). The area under the ROC curve was 0.67 for CRP and 0.64 for YKL-40 (Figure 4). By maximising both sensitivity and specificity for prediction of lung cancer, a cut-point of 2.1 mg l^−1^ was identified for levels of CRP (sensitivity and specificity both 61%) and of 68.6 *μ*g l^−1^ for levels of YKL-40 (sensitivity and specificity both 62%).

### Gastrointestinal cancer

The multifactorially adjusted HR of gastrointestinal cancer for a doubling of YKL-40 levels was 1.37 (95% CI: 1.17–1.61), while further adjustment for CRP levels resulted in a similar HR of 1.36 (1.15–1.60) (Figure 2; likelihood ratio test between models, *P*=0.77). The corresponding HR for a doubling of CRP levels was 1.09 (0.94–1.26), while further adjustment for YKL-40 levels resulted in an HR of 1.02 (0.88–1.19) (Figure 2; *P*<0.001). This means that elevated YKL-40 levels are a risk predictor for gastrointestinal cancer, independent of CRP levels.

The cumulative incidence of gastrointestinal cancer as a function of age increased with increasing levels of CRP and YKL-40 (Figure 3; log-rank, *P*<0.001). Multifactorially adjusted HRs of gastrointestinal cancer were 1.48 (0.95–2.29) for individuals with CRP ⩾1.7 mg l^−1^ and YKL-40 <154 *μ*g l^−1^, 3.36 (1.70–6.64) for individuals with CRP <1.7 mg l^−1^ and YKL-40 ⩾154 *μ*g l^−1^, and 2.41 (1.37–4.24) for individuals with CRP ⩾1.7 mg l^−1^ and YKL-40 ⩾154 *μ*g l^−1^ compared to individuals with CRP <1.7 mg l^−1^ and YKL-40 <154 *μ*g l^−1^ (Figure 3).

The sensitivity and specificity of CRP ⩾1.7 mg l^−1^ was 66 and 49% (Table 2). The corresponding values for YKL-40 ⩾154 *μ*g l^−1^ was 27 and 90% (Table 2). The area under the ROC curve was 0.60 for CRP and 0.68 for YKL-40 (Figure 4). By maximising both sensitivity and specificity for prediction of gastrointestinal cancer, a cut-point of 1.8 mg l^−1^ was identified for levels of CRP (sensitivity and specificity both 54%) and of 69.0 *μ*g l^−1^ for levels of YKL-40 (sensitivity and specificity both 63%).

### Any cancer

Excluding cancers in the categories of lung cancer and gastrointestinal cancer, the major cancers in the category of any cancer were breast cancer (15% of any cancers), prostate cancer (9%), bladder and urinary tract cancer (4%), melanoma (4%), metastases with unknown primary tumour (4%), corpus uteri cancer (3%), cancer of the ovary and female genital organs (3%), and leukaemia (3%).

The multifactorially adjusted HR of any cancer for a doubling of CRP levels was 1.15 (95% CI: 1.07–1.24), while further adjustment for YKL-40 levels resulted in a similar HR of 1.14 (1.05–1.23) (Figure 2; likelihood ratio test between models, *P*=0.26). The corresponding HR for a doubling of YKL-40 levels was 1.09 (1.00–1.20), while further adjustment for CRP levels resulted in an HR of 1.06 (0.96–1.16) (Figure 2; *P*<0.001). This means that elevated CRP levels are a risk predictor for any cancer, independent of YKL-40 levels.

The cumulative incidence of any cancer as a function of age increased with increasing levels of CRP and YKL-40 (Figure 3; log-rank, *P*<0.001). Multifactorially adjusted HRs of any cancer were 1.40 (1.13–1.73) for individuals with CRP ⩾1.7 mg l^−1^ and YKL-40 <154 *μ*g l, 1.54 (0.98–2.43) for individuals with CRP <1.7 mg l and YKL-40 ⩾154 *μ*g l, and 1.62 (1.18–2.23) for individuals with CRP ⩾1.7 mg l and YKL-40 ⩾154 *μ*g l compared to individuals with CRP <1.7 mg l and YKL-40 <154 *μ*g l (Figure 3).

The sensitivity and specificity of CRP ⩾1.7 mg l was 66 and 50% (Table 2). The corresponding values for YKL-40 ⩾154 *μ*g l was 17 and 91% (Table 2). The area under the ROC curve was 0.61 for CRP and 0.62 for YKL-40 (Figure 4). Maximising both sensitivity and specificity for prediction of any cancer, a cut-point of 1.9 mg /l^−1^ was identified for levels of CRP (sensitivity and specificity both 57%) and of 62.0 *μ*g l^−1^ for levels of YKL-40 (sensitivity and specificity both 59%).

## Discussion

In this prospective cohort study of the Danish general population, we observed that elevated YKL-40 levels were associated with an increased risk of gastrointestinal cancer, independently of CRP levels, and that elevated CRP levels were associated with an increased risk of lung cancer, independently of YKL-40 levels. These findings are novel.

The results of this study indicate that the combined use of YKL-40 and CRP does not necessarily improve the prediction ability of either CRP or YKL-40 alone. Although elevated YKL-40 levels were associated with increased risk of gastrointestinal cancer, independently of CRP levels, CRP levels were not related to gastrointestinal cancer risk. These observations mean that YKL-40 alone is enough to predict gastrointestinal cancer risk. A similar issue can be pointed out in the association of CRP and YKL-40 with lung cancer that the present results merely imply that CRP alone is enough to predict lung cancer risk. The complementary and mutually independent associations between CRP and lung cancer, respectively, YKL-40 and gastrointestinal cancer are intriguing. First, although CRP and YKL-40 levels are increased during similar clinical conditions in response to elevated levels of interleukin-6 (Cintin *et al*, 1999; Nordenbaek *et al*, 1999; Vos *et al*, 2000; Ostergaard *et al*, 2002; Pepys and Hirschfield, 2003; Johansen *et al*, 2004, 2009b; Johnson, 2006; Nielsen *et al*, 2011), CRP and YKL-40 are produced by different cell types and in different parts of the body. Thus, CRP is produced in the liver, whereas YKL-40 is produced at the site of pathology by different cells, including cancer cells and cancer-associated macrophages (Pepys and Hirschfield, 2003; Junker *et al*, 2005; Johnson, 2006; Johansen *et al*, 2009b). Second, cancer is not a single entity, but rather a collection of diseases, each characterised by individual pathogenesis, which might be reflected in the plasma.

Although we cannot evaluate the aetiological aspects of an association between CRP, YKL-40, and cancer risk in this observational cohort study of the general population, several biological mechanisms may account for the observed associations. One could speculate that elevated CRP levels closely reflect the extent of pulmonary inflammation. Causes of pulmonary inflammation include smoking, asthma, and chronic lung infections, conditions that are all associated with an increased risk of lung cancer (Engels, 2008). Another cause of pulmonary inflammation is the presence of occult lung cancer (Balkwill and Mantovani, 2001; Coussens and Werb, 2002; Colotta *et al*, 2009). Thus, the association between elevated CRP levels and risk of lung cancer could represent confounding by pulmonary inflammation or reverse causation by occult lung cancer. It seems unlikely that elevated CRP levels play a causal role in the pathogenesis of lung cancer, as three former studies found that single-nucleotide polymorphisms in the *CRP* gene that are associated with increased circulating CRP levels were not associated with an increased risk of lung cancer (Siemes *et al*, 2006; Allin *et al*, 2010; Chaturvedi *et al*, 2010).

As the biological function of YKL-40 is poorly understood and as genetic studies like those mentioned above do not exist for YKL-40, we cannot exclude that YKL-40 could play a causal role in the pathogenesis of gastrointestinal cancer. However, at this stage, we consider it more likely that YKL-40 is a mere marker of gastrointestinal cancer. Thus, chronic gastrointestinal inflammation is a well-known risk predictor for gastrointestinal cancer (Balkwill and Mantovani, 2001; Coussens and Werb, 2002), and YKL-40 levels could closely reflect the amount of gastrointestinal inflammation. Furthermore, in accordance with the observation that gastrointestinal cancers are typically surrounded by extensive inflammation and some fibrosis, we speculate that YKL-40, which is a growth factor of fibroblasts (Recklies *et al*, 2002), is secreted in large amounts by gastrointestinal cancer cells. Thus, the association between elevated YKL-40 levels and increased risk of gastrointestinal cancer could represent confounding by gastrointestinal inflammation or reverse causation by occult gastrointestinal cancer.

Sensitivity and specificity of CRP for predicting lung cancer was both 61% when using a cut-point of 2.1 mg l^−1^, whereas corresponding values for YKL-40 in risk prediction of gastrointestinal cancer were 63% using a cut-point of 69.0 *μ*g l^−1^. Although these values may not seem high, they are in fact comparable to the sensitivity and specificity of low-density lipoprotein cholesterol for prediction of ischemic cardiovascular events (Benn *et al*, 2007).

The interpretation of our data is potentially limited by our amalgamation of heterogeneous cancer types into the three outcome measures lung cancer, gastrointestinal cancer, and any cancer; however, we did not have sufficient statistical power to examine the risk of individual cancer types.

In conclusion, we have demonstrated that elevated YKL-40 levels are associated with increased risk of gastrointestinal cancer, independently of CRP levels and that elevated CRP levels are associated with increased risk of lung cancer, independently of YKL-40 levels.

## Figures and Tables

**Table 1:** Characteristics of participants in the Copenhagen City Heart Study at blood sampling

Participants, no.	8706
Women, no. (%)	4945 (57)
Age at entry, median (IQR) (years)	60 (48–70)
CRP concentration, median (IQR) (mg l^−1^)	1.74 (1.25–3.01)
YKL-40 concentration, median (IQR) (*μ*g l^−1^)	55 (36–90)
Cigarettes smoked per day, median (IQR)^a^	0 (0–14)
	
*Smoking status, no. (%)*	
Never	2252 (26)
Former	2291 (26)
Current	4163 (48)
	
*Alcohol consumption (g per week), no. (%)* b	
⩽168 (women) or ⩽252 (men)	7395 (85)
>168 (women) or >252 (men)	1311 (15)
	
*Body mass index (kg* *m*^*−2*^*), no. (%)*	
<18.5	165 (2)
18.5–24.9	4146 (48)
25–29.9	3123 (36)
⩾30.0	1272 (15)
	
No. of first lung cancers	302
No. of first lung cancers within the first 5 years after blood sampling	85
No. of first gastrointestinal cancers	434
No. of first gastrointestinal cancers within the first 5 years after blood sampling	130
No. of first any cancers	1453
No. of first any cancers within the first 5 years after blood sampling	479

Abbreviation: IQR=interquartile range.

a Smoking of other types of tobacco were converted to cigarette equivalents.

b Cutoff values concerning alcohol consumption are based on the recommendation of the Danish National Board of Health.

**Table 2:** Sensitivity and specificity of CRP and YKL-40

	**Sensitivity (%)**	**Specificity (%)**
*Lung cancer*		
High CRP	79	49
High YKL-40	19	90
High CRP, high YKL-40	12	95
		
*Gastrointestinal cancer*		
High CRP	66	49
High YKL-40	27	90
High CRP, high YKL-40	8	95
		
*Any cancer*		
High CRP	66	50
High YKL-40	17	91
High CRP, high YKL-40	8	96

Abbreviation: CRP=C-reactive protein.

High CRP: CRP ⩾1.7 mg l^−1^.

High YKL-40: YKL-40 ⩾154 *μ*g l^−1^.
