# Supplementary material for: Cancer risk by combined levels of YKL-40 and C-reactive protein in the general population
Source: Br J Cancer. 2011 Nov 17;106(1):199–205. doi: 10.1038/bjc.2011.501 (PMC3251851; doi:10.1038/bjc.2011.501)
Supplement: Supplementary Information [file bjc2011501x1.doc]

**Supplemental Data**

**Supplemental Figure 1. Risk of cancer for a doubling of CRP or YKL-40 levels in the general population.**

Circles depict hazard ratios and vertical lines depict 95% confidence intervals for a doubling of CRP levels (open circles) and YKL-40 levels (full circles). Hazard ratios were multifactorially adjusted for age, sex, smoking, alcohol consumption, and body mass index.

| **Supplemental Table 1. Risk of cancer by CRP and YKL-40 levels in the general population** | | | | | |
| --- | --- | --- | --- | --- | --- |
|  | **No. of participants** |  | **No. of events** |  | **HR (95% CI)** |
| **Lung cancer** |  |  |  |  |  |
| Doubling of CRP levels | 8688 |  | 302 |  | 1.15 (1.05 to 1.26) |
| Doubling of YKL-40 levels | 8688 |  | 302 |  | 1.11 (0.99 to 1.24) |
|  |  |  |  |  |  |
| CRP<1.7mg/L, YKL-40<154μg/L | 3993 |  | 95 |  | 1.00 |
| CRP≥1.7mg/L, YKL-40<154μg/L | 3827 |  | 164 |  | 1.41 (1.09 to 1.82) |
| CRP<1.7mg/L, YKL-40≥154μg/L | 244 |  | 9 |  | 1.06 (0.53 to 2.11) |
| CRP≥1.7mg/L, YKL-40≥154μg/L | 624 |  | 34 |  | 1.66 (1.11 to 2.49) |
|  |  |  |  |  |  |
| **Gastrointestinal cancer** |  |  |  |  |  |
| Doubling of CRP levels | 8623 |  | 434 |  | 1.02 (0.94 to 1.12) |
| Doubling of YKL-40 levels | 8623 |  | 434 |  | 1.16 (1.05 to 1.27) |
|  |  |  |  |  |  |
| CRP<1.7mg/L, YKL-40<154μg/L | 3966 |  | 150 |  | 1.00 |
| CRP≥1.7mg/L, YKL-40<154μg/L | 3797 |  | 213 |  | 1.17 (0.95 to 1.46) |
| CRP<1.7mg/L, YKL-40≥154μg/L | 240 |  | 24 |  | 1.81 (1.17 to 2.82) |
| CRP≥1.7mg/L, YKL-40≥154μg/L | 620 |  | 47 |  | 1.46 (1.03 to 2.05) |
|  |  |  |  |  |  |
| **Any cancer** |  |  |  |  |  |
| Doubling of CRP levels | 8262 |  | 1453 |  | 1.05 (1.01 to 1.11) |
| Doubling of YKL-40 levels | 8262 |  | 1453 |  | 1.07 (1.02 to 1.13) |
|  |  |  |  |  |  |
| CRP<1.7mg/L, YKL-40<154μg/L | 3852 |  | 551 |  | 1.00 |
| CRP≥1.7mg/L, YKL-40<154μg/L | 3619 |  | 722 |  | 1.14 (1.02 to 1.28) |
| CRP<1.7mg/L, YKL-40≥154μg/L | 226 |  | 55 |  | 1.31 (0.99 to 1.74) |
| CRP≥1.7mg/L, YKL-40≥154μg/L | 565 |  | 125 |  | 1.27 (1.04 to 1.56) |

Data are from the Copenhagen City Heart Study, in which participants were followed for up to 18 years for development of cancer. Plasma levels of CRP and YKL-40 were measured at study entry. Hazard ratios were multifactorially adjusted for age, sex, smoking, alcohol consumption, and body mass index.

HR=hazard ratio; CI=confidence interval.
